# 

**Published:** 1897-04

**Authors:** 


					﻿Vol. XVII.
APRIL, 1897.
NO. 4.
JUHE
pOMtEOPATHIC PHYSICIAN,
A Monthly Journal of Homoeopathic Materia Medica
and Clinical Medicine.
“ He is a freeman whom truth makes free, and all are slaves beside.”
“Seek the Truth: come whence it may, cost what it will.”
EDITED AND PUBLISHED BY
WALTER Nl. JAMES, M. D.
PHILADELPHIA :
Ko. 1231 LOCUST STREET.
LONDON:
ALFRED HEATH & CO., Sole Agents for Great Britain,
114 Ebury Street, Eaton Square.
Yearly Subscription, $2.50, invariably in advance. Single numbers, X5 ets.
Entered at the Philadelphia Post-Office aa Second-Class Mail Matter.
				

